# Automatic Annotation of Change Detection Images

**DOI:** 10.3390/s21041110

**Published:** 2021-02-05

**Authors:** Nathalie Neptune, Josiane Mothe

**Affiliations:** 1 Université fédérale de Toulouse, Université Paul Sabatier, 31062 Toulouse, France; 2 Université fédérale de Toulouse, Université Jean-Jaurès, INSPE, 31058 Toulouse, France; josiane.mothe@irit.fr; 3 Institut de Recherche en Informatique de Toulouse, UMR5505 CNRS 118 Rte de Narbonne, CEDEX 09, 31062 Toulouse, France

**Keywords:** binary satellite image change detection, multimodal learning, automatic satellite image annotation

## Abstract

Earth observation satellites have been capturing a variety of data about our planet for several decades, making many environmental applications possible such as change detection. Recently, deep learning methods have been proposed for urban change detection. However, there has been limited work done on the application of such methods to the annotation of unlabeled images in the case of change detection in forests. This annotation task consists of predicting semantic labels for a given image of a forested area where change has been detected. Currently proposed methods typically do not provide other semantic information beyond the change that is detected. To address these limitations we first demonstrate that deep learning methods can be effectively used to detect changes in a forested area with a pair of pre and post-change satellite images. We show that by using visual semantic embeddings we can automatically annotate the change images with labels extracted from scientific documents related to the study area. We investigated the effect of different corpora and found that best performances in the annotation prediction task are reached with a corpus that is related to the type of change of interest and is of medium size (over ten thousand documents).

## 1. Introduction

An increasing number and variety of Earth observation (EO) satellites are orbiting our planet providing a wealth of data for those who need to perform environmental monitoring at various scales. This is particularly useful for the monitoring of large or very remote areas where on-site data acquisition is impractical. Indeed, the impact of environmental events such as deforestation [1,2], wildfires [3], and other natural disasters [4,5] can be assessed with data from EO satellites. With change detection techniques, the various changes that are happening on the Earth’s surface can be automatically detected by analyzing images of a given area taken at different times [6]. Such techniques have been used to monitor loss and disturbances in forests [2,7], to track change in urban areas [8], and also to map out areas affected by natural disasters [4,5]. When a change detection method neither provides nor needs additional semantic information beyond the change/no-change pixels, it is called a binary change detection method. Deep learning has been used for binary change detection in urban areas and forests [8,9,10], and it has been shown to provide improved results compared to traditional change detection methods while requiring less post-processing [9,10].

Annotation, or labeling, is needed to add semantic information to EO data. In fact, EO images without ground truth labels (or annotations) are plentiful. The American Landsat (https://www.usgs.gov/land-resources/nli/landsat) and European Copernicus (https://www.copernicus.eu/en) programs, for instance, provide free access to the images produced by their respective satellite missions with new images made available every day. Hence, detecting changes that have occurred in an area of interest at a specific time might require the use of satellite images that have not yet been annotated. In the absence of semantic labels, changes can still be detected by comparing the images; however, semantic information about those changes will be missing. Without this information it is not possible to tell anything more about the area of interest beyond the fact that some change was detected at specific locations. Having labels or annotations for each image, acquired either through human (expert) annotators or automatic methods, solves this problem. In a context where expert annotators are not available, and few or no annotated images exist for an area of interest, automatic annotations can fill the gap.

Supervised machine learning has been successfully used for semantic change detection [8]. Such models are trained on images along with their semantic change masks. In the case of deep learning models in particular, the scale of the data needed for training makes it impractical to have experts manually annotate all the images. Crowdsourcing has been used to provide image annotations at very large scale [11]. A similar approach is not well suited for EO images because some expertise might be required to properly identify and differentiate among classes. As a result, automatic and semi-automatic approaches are commonly used to build large labeled EO data sets [1,7].

Scientific literature published by researchers who work with EO images is undoubtedly a source of expert knowledge in the field. Publications in Earth sciences therefore can be seen as a very large source of expertise that could be leveraged for interpreting EO images. Furthermore, it is available at a large scale. In fact, across all scientific disciplines, the number of publications has grown exponentially in the past decades [12]. Using the text from those publications, we can train a neural network to learn word vector representations or embeddings. Word embeddings are vectors of real numbers, which are learned by a neural network in an unsupervised way, from a text corpus, without any annotation. For example, Word2vec [13] learns word vectors with the skip-gram model. With Word2vec, the words with similar meaning will have a similar vector representation.

With visual semantic embeddings, we learn to represent textual and visual data in the same vector space. The closer those data points are semantically, the smaller their distance is in this joint space. Several approaches have been proposed including the joint embedding of images and words into a common low-dimension space [14] for image classification, and the embedding of images and sentences into a common space for image description [15]. By using joint image and text embeddings we propose to automatically assign relevant annotations to image pairs where changes are detected. Therefore, we perform two core tasks: change detection and the annotation of the image pairs. We propose to use scientific publications as a source of annotations, for which we learn the vector representations using a neural language model. These annotations can be used in subsequent tasks such as image indexing and retrieval. While change detection can be applied to any type of image pairs of the same scene or location, we are focusing on the case of changes occurring in forest areas to test our approach.

## 2. Materials and Methods

### 2.1. Portugal 2017 Forest Fire Dataset

We used a dataset of images from June and July 2017 of the area of Pedrógão Grande in Portugal, which was affected by wildfires in June 2017. The images were captured by the MultiSpectral Instrument of the Sentinel-2 satellite. The first image is from 14 June 2017, and the second image is from 4 July 2017. Both images are from the Sentinel-2 tile T29TNE. In this work, we used the three red, green, and blue (RGB) spectral channels, which are the B4, B3, and B2 bands, respectively, for Sentinel-2. The data, provided by the European Space Agency (https://www.esa.int/) (ESA), were preprocessed and therefore atmospherically corrected and resampled at 10 m. These images are openly available for download from the Copernicus Open Access Hub (https://scihub.copernicus.eu/).

For the ground truth, the Normalized Burn Ratio (NBR) [16] was used to identify the burnt areas and label the pixels accordingly to obtain a binary segmentation mask. The Normalized Burn Ratio is presented as a reliable means to detect burnt areas by comparing a first pre-fire image to a second post-fire image. The NBR is defined as the ratio between the Near Infrared (*NIR*) and the Shortwave Infrared (SWIR) spectral bands, (NIR−SWIR)/(NIR+SWIR). The difference between the two images in terms of NBR decreases with time because of vegetation regrowth. In our case, the image of the burnt areas was taken only about two weeks after the fires, which limits regrowth. Furthermore, the burnt areas are mostly large continuous areas, and a visual evaluation of the performance of the NBR can also be performed.

**Figure 1:**
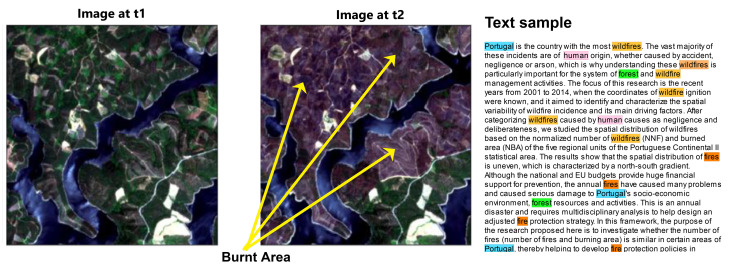
Sample images from the dataset with an example text containing relevant keywords for annotating the images. A pair of images is shown with the first image taken at t1 (14 June 2017), and the second one taken at t2 (4 July 2017) showing apparent burnt areas. A portion of text is shown to illustrate the content of the scientific publications being used, and relevant keywords within the text are highlighted.

We collected a total of 16 publications, titles, and abstracts from the Web of Science (https://www.webofknowledge.com), which were published from 2017 to 2019 using the topic “Portugal forest fire June 2017”. As there have been other instances of wildfires in the country, we restricted our corpus to the documents more closely related to the June 2017 events. Figure 1 shows sample image patches from the dataset from before and after the wildfires along with a text sample with the most relevant keywords highlighted as candidate keywords for the annotations. Because the number of publications that match exactly our dataset is small, whereas word embedding models are better trained on large corpora, we also used a related corpus [17], initially created for investigating deforestation in scientific literature, containing 16,136 publications from the years 1975 to 2016. While this larger corpus does not exactly match our event of interest, it is nevertheless thematically related to it and is appropriate to train a word embedding model.

### 2.2. Change Detection Methods for Image Pairs

Our approach for annotating the changes in satellite images uses a change detection method for image pairs. We are therefore using a bi-temporal approach to change detection where we only consider two images of the same area taken at two different times, once before the change event occurred and once after the change event occurred. Indeed, the first step to adding semantic annotations to changes in EO images is to locate the changes within the images and consequently identify the image pairs with changes in them.

Binary change detection methods allow systems to detect if and where a change occurred, whereas semantic change detection methods also specify semantic information about the change that is detected [18]. Both types of methods have been applied in the literature to satellite images to detect land cover changes with the most recent ones using deep learning models [8,19]. Daudt et al. [8] proposed a change detection deep learning model for satellite images based on U-Net [20], which is an encoder–decoder architecture with skip connections between the encoding and decoding streams. The U-Net architecture was initially proposed for the segmentation of biomedical images. Three variations of the model were proposed, the first variation performing early fusion by taking the concatenation of the images as input, effectively treating them as different color channels. In this case, the change detection problem is posed as an image segmentation task with two classes: change and no-change. The second and third model variations are siamese versions of the U-Net architecture where the encoder part is duplicated to encode each image separately, and skip connections are used in two ways, by concatenating either the skip connections from both encoding streams or the absolute value of their difference. Another variation of U-Net with early fusion, for change detection, which uses dense skip connections was proposed by [19].

In this work, we used a model similar to the early fusion model proposed by [8] to perform the binary change detection task. It is the most simple and generic architecture that outperformed the siamese models on the binary change detection task when only the 3 RGB channels were used [8]. In our case we are only using the RGB channels.

### 2.3. Visual Semantic Embeddings

Our automatic annotation approach for change image pairs uses a visual semantic embedding model to match the images with the relevant keywords extracted from the publication corpus. When the semantic information about the classes present in EO images is inconsistent or lacking, different solutions have been proposed to extract that information from other sources such as ontologies [4] or geo-referenced Wikipedia articles [21]. Another solution is to use visual semantic embeddings by representing the images and text in the space vector space and learning the classes of the unlabeled images based on the similarity between the vector representations across the image and text modalities [14]. In this common vector space the unlabeled images are close to the text of their labels.

By integrating an ontology to the segmentation process of pre- and post-disaster images, authors in [4] showed that overall accuracy went from 67.9% to 89.4% for images of their test area. With a reduced number of samples (200), authors in [21] demonstrated that using Wikipedia annotations for the task of semantic segmentation, the Intersection-over-Union (IoU) score was 51.70% compared to 50.75% when pre-training on ImageNet. In both cases, the methods were tested on images of urban areas. While the use of the ontology created by experts in [4] improved greatly the accuracy of the classification algorithm, it came at the high cost of expert hours. The crowdsourcing approach using Wikipedia data in [21] while promising, resulted only in modest improvement for the semantic segmentation task. In our case, the scientific publications written by researchers can be seen as both a source of expert knowledge and a crowdsourced resource as they are coming from a large number of scientists.

**Figure 2:**
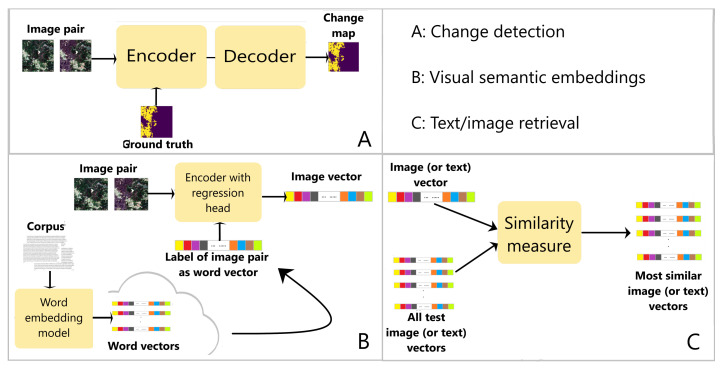
Overview of our method for annotating an image pair with words extracted from a corpus of scientific publications. An encoder–decoder model is used to predict a change map for an image pair (**A**). A word embedding model is used to learn the vector representations of all the words in a corpus; a regression head is added to the encoder used in A, to predict a vector for the image pair, based on the word vector corresponding to its label, which is used as the ground truth (**B**). The proposed method is tested by performing text and image retrieval tasks with the predicted vectors (**C**). The vector of an image pair, referred to as an image vector, is compared to all the word vectors learned from the corpus in B, using a similarity metric; the vector of the label of an image pair, referred to as a text vector, is compared to all the predicted image vectors using the similarity metric. When the input is an image vector, the most similar text vectors are found. When the input is a text vector, the most similar image vectors are found.

We propose to use expert knowledge through relevant scientific papers from which annotations are extracted. Our method therefore performs change detection, in satellite image pairs by predicting change pixels, and it also performs semantic annotation of the image pair by predicting its labels. We used a deep learning network architecture based on U-Net [20], and we tested our method on the data described in Section 2.1. Figure 2 shows an overview of our method.

### 2.4. Combining Change Detection and Visual Semantic Embeddings

Our approach is built on visual semantic embeddings for annotating changes to add semantic labels to binary change detection. We used deep learning models to predict the binary change map and the vector representation of the images in the word vector space. The U-Net [20] encoder–decoder architecture was used. A regression head was added on top of the encoder to learn the feature vector of the image pair in the same dimension as the vector of its label (Figure 2), using an approach similar to [14]. In [14], the vector representations of images are projected into vectors of the same dimensions as the word vectors, and the model predicts the label vector using a similarity metric.

We used a text corpus made of publications related to the area and the type of change of interest to train a word embedding model. We used the word embedding of the image labels to train the regression head. When making predictions, we searched for an annotation among all the word embeddings learned by the word embedding model. Predicted annotations can, therefore, be among labels present during training, but they might also be among words that have not been seen during training but are nearest neighbors of the image label in the word vector space. We used Fasttext [22] as the neural language model to learn the word embeddings. Fasttext [22] is an extension of Word2vec [13]. With Fasttext, words are broken into n-grams, which are portions of words. For example, the word “forest” will have 5-grams such as “fores” and “orest”. Each n-gram will have its own vector, and the full word will have a vector that is the sum of all its n-gram vectors. We used different corpora to investigate how results might differ with a larger, more general text corpus as opposed to a smaller, more relevant corpus. In addition to the Portugal Forest Fire corpus, we used word embeddings trained on a forest-related corpus from [17] and on Wikipedia. To keep the Wikipedia and forest embeddings relevant to our area of study we aligned them to our corpora embeddings using [23].

Our approach differs from [24] in that we perform change detection with a deep learning model, and our word vectors are trained on a specialized corpus of scientific documents. This is intended to make our model more scalable and provide more relevant annotations. Unlike [8,19], our approach is suitable for a change detection dataset that is not fully annotated, meaning that some annotations may be missing or incorrect. We are also not manually building an ontology like [4,25]. Our approach is closer to [21]; however, they do not perform the change detection task but classify and segment individual images.

We want to have a model that is suitable for environmental applications; therefore, we tested on these types of data first. We tested on optical satellite imagery, but our proposed approach can be adapted to other types of remotely sensed data including 3D point clouds, with an adapted network architecture. Additionally, applications in domains other than environmental sciences are possible, as long as we can find a dataset of image pairs with a corpus of relevant documents. We assessed the task of automatically annotating a pair of EO images used in change detection. Given a pair of images without annotations, we want to automatically predict the correct labels for it. Labels are deemed correct if they match the ones assigned by the human annotator. We trained the visual semantic model with a single label per image pair and treated this as a single-label multiclass classification problem. We used the cosine similarity to measure the similarity between the predicted annotations and target annotations. We reported the recall at *k*, which is commonly used for text-image/image-text retrieval tasks [26], to evaluate the visual semantic embeddings. For the purpose of adding semantic information to the change detected in the images, we also looked at predicted annotations that were not an exact match with the target label but were among its closest word vectors.

## 3. Results

We tested our method on a dataset of images from the Sentinel-2 mission MultiSpectral Instrument (MSI) taken over an area in central Portugal before and after wildfires erupted in June 2017. This dataset is described in detail in Section 2.1.

We performed our two core tasks of binary change detection and annotation of change images with deep learning. The change detection model used is a fully convolutional neural network (U-Net) [20] with a residual network (ResNet) [27] encoder. For learning the visual semantic embeddings needed for our annotation task we used a convolutional neural network encoder with a regression head. The regression head is a small neural network added on top of the encoder. This network is made of two fully connected layers with batch normalization, dropout regularization at 25% then 50%, and rectified linear activation function (ReLU). It takes the output of the encoder as its input, then applies adaptive max pooling to reduce the number of dimensions, then flattens the resulting tensor, then passes it through the linear layers, and outputs a vector of dimension 300 which is the same size as the word vectors. We report the evaluation of the change detection task (Table 1) and the annotation task of the image pairs with visual semantic embeddings (Table 2).

We used two training strategies. The first strategy was to use the encoder to perform each task independently, once for the change detection and once for the visual semantic embedding learning. The second strategy was first to train the model for the binary change detection task, and then use that trained model to train it on the visual semantic embedding learning. In both strategies we performed the change detection task first. We report the results of our experiments in the following sections.

### 3.1. Binary Change Detection

The binary change detection task was treated as a binary image segmentation task where two images are segmented as a pair. We trained a U-net [20] with pairs of images of the same area taken at different times, as input, and the segmentation map (positive or negative label) of the pixels as ground truth. The images were concatenated and passed to the network as a single six-channel input. The output was the predicted segmentation map. Pixels that were positive in the segmentation map are the pixels where change occurred. The model was thus trained to learn to differentiate between positive and negative pixels in the image pair.

We used the segmentation models implemented by [28] with Pytorch [28] version 1.6.0 and Python version 3 to train the model with binary cross-entropy loss function and Adam optimizer with default parameters. We trained each model for 200 epochs with a learning rate at 0.0001. We trained the network from scratch without any pretraining. We report the precision, recall, F1 score and mean Intersection over Union (mIoU) obtained from training the U-net model with residual network encoders (ResNet) [27] and very deep convolutional networks originally from the Oxford Visual Geometry Group (VGG) [29], which are among the state-of-the-art convolutional neural networks for feature extraction from images. Table 1 shows the results for binary change detection on the images. The values of the F1 scores were all between 0.80 and 0.83. For the mIoU, the values were between 0.67 and 0.70. While the overall performance varied with each network, they remained comparable.

On the basis of results from the binary change detection task, we chose a ResNet34 encoder for the visual semantic embedding learning task. While it did yield slightly lower F1 and mIoU scores than the VGG models, we found it slightly faster to train in our experiments.

**Figure 3:**
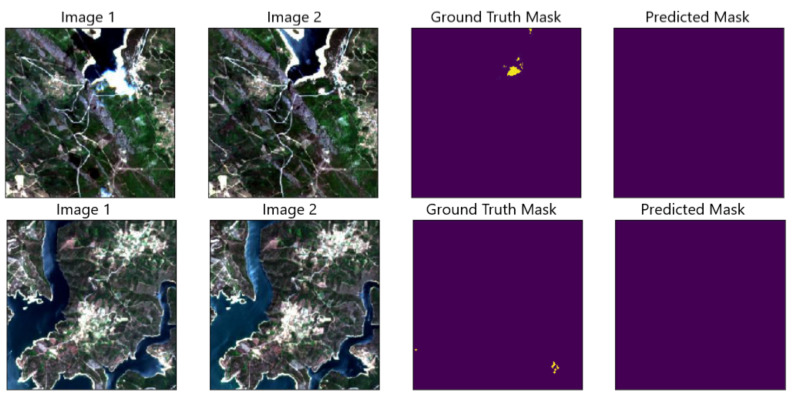
The model correctly predicts negative pixels inside water bodies when the ground truth mask has them marked as positive. For these image pairs, the model does not make the same mistake as the ground truth mask, which erroneously marked parts of the water as burnt vegetation.

In terms of qualitative results, we found that in some cases the model did better than the ground truth mask at predicting negative pixels. In fact, in some cases, the Normalized Burn Ratio used to create the ground truth mask erroneously marked pixels inside water bodies as burnt vegetation. The deep learning model seemed to be less prone to make the same mistake, as illustrated in Figure 3.

### 3.2. Annotation of Image Pairs with Visual Semantic Embeddings Using ResNet34 Encoder

The goal of the visual semantic task is to find a common representation for images and text in which the images and texts that are related are similar. With this common representation, we can then find words “similar” to the images that we wish to annotate and choose the needed annotations from those words. Given an image pair, we learn the vector representation of that pair in the word vector space. We do this by performing regression with the encoder used for the binary change detection task. We add additional layers to the encoder (a regression head) to predict a single vector for the image features. This image feature vector is of the same dimension as the word vector for the label of the image pair, which we obtained from FastText [22]. The images are labeled on the basis of their land cover and land use classes. A total of six unique label values were defined: ‘agriculture’, ‘city’, ‘forest’, ’ground’, ‘wildfire’, and ‘water’. Each image pair had one or several labels, based on its content. We used one label per image pair to test our method. The label was selected as follows: if wildfire was detected, the image pair was labeled with ‘wildfire’, if not it was labeled with one of its other labels. The input of the model is the image pair similarly to the change detection task, the difference is that the ground truth is now a word vector, and the training objective is to maximize the similarity between the word vector and the image vector. We used cosine similarity as the vector similarity metric that we are trying to maximize.

We applied our two training strategies with the different combinations of word embeddings. In addition to word embeddings from our corpus (PF) (Section 2.1), we also tested our method with pretrained Wikipedia (Wiki) word embeddings from FastText [22] as is common in the state of the art. Additionally, we tested our method with a forest-related corpus (Forest) from [17] to find out if having a relatively big corpus, which is more thematically close to our images than Wikipedia, will lead to better predictions. The results can be found in Table 2.

In Table 2 and Table 3, the training strategy indicates whether the model was first pre-trained on the change detection task or not. Aligned word vectors are noted with the ‘&’ symbol, meaning the vectors on the left were aligned to the vectors on the right using [23]. We reported the average cosine similarity (CosSim) of each model, which is the average of the angle between the predicted vectors and the ground truth vectors. We calculated annotation retrieval metrics to evaluate the performance of our visual semantic model as is common for visual semantic learning models [14,26]. For each ground truth annotation, we found the images from the test dataset that were most similar to it using the k-nearest neighbors algorithm. We reported the average R-precision (R-Prec), where *R* represents the number of image pairs with a given annotation, and $A_{c}$ is the number of correctly predicted annotations; the R-precision is given by $A_{c}/R$. For each annotation, the R-precision is therefore the proportion of top R image pairs that were correctly found to match this label, based on the similarity between the predicted vector of the image pair and the vector of the label. R is the total number of image pairs with that label in the dataset. The value reported in Table 2 is the average R-precision over all tested keywords for each model. Finally, we reported the average recall at *k* (R@k) with *k* taking values 1, 5, and 10. The recall at *k* is calculated for text-image retrieval where the query is an annotation, and the result is the corresponding image pairs; for image-text retrieval where the image pair is the query, the result is the corresponding text. For a given text/image (in our case annotation/image pair), the recall at *k* was set to 1 if the target text/image was present in the top k-nearest neighbors and 0 if not. For the image-text retrieval task, the recall at 1 was equal to the R−precision in our case, because for each image pair we only had a single annotation as its ground truth.

## 4. Discussion

From the results, in Table 2, we can see that while the average cosine similarity for the model trained on the PF database was the highest, this model reached the worst performance overall on both image and text retrieval. The high cosine similarity seems to indicate that the network learned image vectors that were very similar to their corresponding word vectors, which might be seen as an indication that it will be able to predict annotations well. This was not the case. We think that due to the limited size of this corpus it is likely that it simply did not learn enough relevant vectors to be able to correctly match the images with their annotations, in the retrieval tasks.

For image retrieval evaluation, when the query is a word and the result is an image pair, the highest recall at 1, on average, is obtained when the network is not pre-trained. The network trained on the Forest corpus, without pre-training, has the highest recall at 1 for image retrieval. Pre-training the network on the change detection task slightly improves R-precision and recall at 10 for image retrieval, on average. On average recall at 1 decreases when the network is pre-trained on the change detection task. This might be due to the fact that the ground truth annotations do not always capture the differences between the two images, which is essentially what the change detection task does. In fact, for most image pairs, there is no difference to be found. It is likely that by emphasizing the features related to change in the image pair, the pre-training resulted in lower performance for the retrieval task when there are no changes in the image pair.

**Figure 4:**
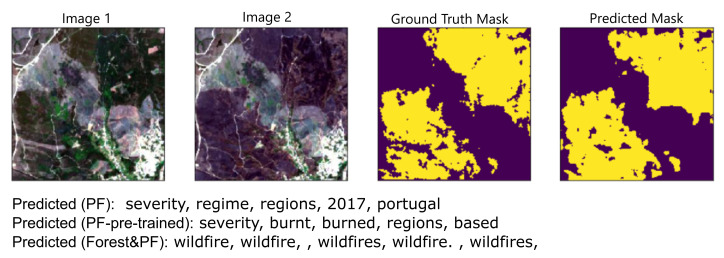
An image pair with its ground truth mask and the mask predicted by the change detection model (Section 3.1) along with the top five annotations predicted by three models. The results from the models trained on PF without pre-training, PF under the change detection pre-training strategy and Forest aligned with PF are shown.

For text retrieval evaluation, when the query is an image pair and the result is a word, considering only recall at 1, the models trained with the corpora that were aligned with the PF corpus obtained the best results in the no-pretraining strategy, the difference for Wiki & PF is the highest of the two, at 0.13. For the pre-training strategy, the model trained on the Forest corpus reached the best recall at 1 of 0.55; however, it performed less well than the top performers under the strategy, without pre-training, for this same task, where the highest recall at 1 was 0.57. The text retrieval results show one limitation of our approach, as we do not perform any re-ranking or merging of the predicted annotations, we simply use the nearest neighbors; this results in many variations of the same word in our predicted annotations in many cases (Figure 4). One area of improvement would be to post-process our results and filter out words that are only variations of the same word or synonyms.

Pre-training on the change detection task can be beneficial when using aligned corpora for training the visual semantic embeddings. It increases the recall at 1, from 0.25 to 0.50 for Wiki & PF, and recall at 5, from 0.50 to 0.75 for Forest & PF, for image retrieval.

Models trained with words from the PF corpus, which is the most related to our images, perform less well than the other model in almost all metrics. We can try to qualitatively evaluate samples of the predictions made by these models to see whether they could still be used to add semantic information to the changes detected in the images. As shown in Figure 4, while models trained on the PF corpus failed to predict the correct image-pair annotation, they had related words in their top five predictions that add semantic information to the images. Additionally, the effect of the pre-training can be seen in the differences between the top words predicted by each model. The model with change detection pre-training is predicting “burn”-related words, whereas the model that was not pre-trained is not. The third model which was trained on the Forest corpus aligned with the PF corpus predicts the image annotation correctly; however, all the other predictions in the top five, for that model, are variations of that same annotation, and no additional information can be learned from them. These observations lead us to presume that a corpus that is very thematically related to the images is likely to predict annotations that add semantic information, but in order to predict the correct top annotation this corpus should not be too small. We tested to see if the model can learn the annotations when it is trained only on a single (post-change) image. We found, in that case, the model performs slightly better in the image retrieval tasks, where the goal is to predict an image for a given annotation. However, it underperforms in text retrieval tasks, where the goal is to predict an annotation for an image (Table 3). Finally, adding some post-processing, for example, merging synonyms, might improve the quality of the results in general.

## 5. Conclusions

We set out to find a way to predict relevant annotations for pairs of satellite images in areas undergoing visible change that can be detected by comparing the two images. Our goal is to have a model that can annotate unlabeled image pairs, and then be able to provide additional semantic information on the area of interest as extra annotations. We demonstrated that this can be done using state-of-the-art deep neural networks. Since both tasks rely on feature extraction from the images, they can share the same feature extractor, i.e., the same convolutional neural network encoder. We attempted to use word vectors learned from a corpus relevant to the area and the changes of interest to predict the most relevant keywords. We showed the limitation of this basic approach with image-text retrieval metrics and demonstrated how, using a larger, less relevant corpus that is aligned to the initial small corpus, we can achieve better performances on the retrieval tasks, in particular in the image to text retrieval task, which is in essence what annotation prediction is doing (i.e., given an image predict the top words associated with it). To the best of our knowledge, this is the first work to propose a method for learning to predict annotations of change detection image pairs, with word vectors learned from a scientific publication corpus. One limitation of this work is the size of the dataset used. We wanted to demonstrate how our method works on real data; therefore, we used satellite images from an area with real change events and a corpus of scientific documents related to both the area and the changes that occurred there. Our proposed method can be applied to a dataset of images presenting several types of changes. The only requirement, for this dataset is that the image pairs used for training are labeled with their respective change type, similarly to the dataset that we used in this work. For the corpus, any collection of documents with text related to the types of changes of interest can be used. Our approach can be used as is, or easily adapted to other types of input data including from other types of sensors such as radar. There has been increasing interest in using 3D data for environmental applications such as change detection. However, like most data captured by remote sensing, these 3D data are mostly unlabeled. Our approach could be used to label 3D data with an adapted encoder. In this work, we demonstrated the feasibility of our proposed method. A future direction for our work is to apply the same approach to larger time series of satellite images, and use different types of pre-training of the network. Future studies could also continue to explore broader applications of our method, for example, to satellite image datasets which are larger and more diverse, in terms of types of changes, than the dataset we used in this work. Along with these new datasets, corresponding corpora, containing not only the titles and the abstracts of the publications but also their whole text, could improve the annotation learning. Further work is certainly required to more deeply combine the two tasks of predicting change and predicting annotations in order for each task to help improve the other.

## Figures and Tables

**Table 1:** ResNet and VGG encoders yield comparable performance measures for the binary change detection task. The ResNets slightly outperform the VGGs in precision, the VGGs slightly outperform the ResNets in recall, and they have higher F1 and mIoU scores overall.

Encoder	Precision	Recall	F1	mIoU
ResNet18	0.79	0.80	0.80	0.66
ResNet34	0.78	0.85	0.81	0.69
ResNet50	0.77	0.85	0.81	0.68
VGG11	0.77	0.88	0.82	0.70
VGG16	0.76	0.88	0.82	0.69
VGG19	0.79	0.84	0.82	0.69

**Table 2:** Choice of corpus and training strategy both influence the performance of the visual semantic model. Recall at 1 is higher on average for image retrieval when the network is not pre-trained. Pre-training the network on the change detection task slightly improves R-precision and recall at 10 for image retrieval, on average, when the query is a word and the result is an image pair. The models trained with the corpora that were aligned with the PF corpus obtained the best results in the no-pre-training strategy. For the pre-training strategy, the model trained on the Forest corpora reached the best recall at 1; however, it performed less well than the top performers under the no-pre-training strategy for this same task.

			Image Retrieval				Text Retrieval		
**Training Strategy**	**Word Vectors**	**CosSim**	**R-Prec**	**R@1**	**R@5**	**R@10**	**R@1**	**R@5**	**R@10**
No Pre-training	PF	0.99	0.17	0.25	0.25	0.25	0.00	0.10	0.29
	Forest	0.70	0.36	0.75	0.75	0.75	0.47	0.82	0.90
	Wiki	0.75	0.39	0.50	0.75	0.75	0.50	0.68	0.72
	Forest & PF	0.71	0.40	0.25	0.50	0.75	0.57	0.61	0.61
	Wiki & PF	0.74	0.30	0.25	0.75	0.75	0.57	0.69	0.69
	Wiki & Forest	0.75	0.34	0.25	0.75	0.75	0.51	0.56	0.56
Pre-training	PF	0.99	0.18	0.00	0.50	0.50	0.00	0.04	0.18
	Forest	0.66	0.27	0.25	0.50	0.75	0.55	0.65	0.68
	Wiki	0.69	0.40	0.50	0.75	0.75	0.50	0.68	0.72
	Forest & PF	0.70	0.36	0.25	0.75	0.75	0.52	0.60	0.63
	Wiki & PF	0.69	0.37	0.50	0.75	0.75	0.44	0.45	0.45
	Wiki & Forest	0.70	0.40	0.50	0.75	0.75	0.51	0.51	0.51

**Table 3:** Training the visual semantic model only on the second (post-change) image yields lower values for recall at *k* for the text retrieval (annotation prediction task), but it improves image retrieval scores. When the same visual semantic embedding model was trained on a single image taken after the change event, it reached lower scores than when trained on the image pair, for all the corpora, in text retrieval. The model performs better in image retrieval than when trained on the image pair.

			Image Retrieval				Text Retrieval		
**Training Strategy**	**Word Vectors**	**CosSim**	**R-Prec**	**R@1**	**R@5**	**R@10**	**R@1**	**R@5**	**R@10**
No Pre-training	PF	0.99	0.19	0.25	0.50	0.50	0.00	0.00	0.00
	Forest	0.63	0.42	0.75	0.75	0.75	0.42	0.54	0.54
	Wiki	0.70	0.41	0.50	0.75	0.75	0.47	0.59	0.61
	Forest & PF	0.62	0.42	0.25	0.75	0.75	0.40	0.41	0.41
	Wiki & PF	0.69	0.37	0.25	0.75	0.75	0.39	0.49	0.49
	Wiki & Forest	0.72	0.40	0.50	0.75	1.00	0.49	0.53	0.54

## Data Availability

Publicly available datasets were analyzed in this study. This data can be found at: https://scihub.copernicus.eu/dhus/#/home, http://apps.webofknowledge.com/, and https://fasttext.cc/docs/en/pretrained-vectors.html.
